# Presence of Systemic Inflammatory Response Syndrome Predicts a Poor Clinical Outcome in Dogs with a Primary Hepatitis

**DOI:** 10.1371/journal.pone.0146560

**Published:** 2016-01-25

**Authors:** Scott Kilpatrick, Margaret Dreistadt, Polly Frowde, Roger Powell, Elspeth Milne, Sionagh Smith, Linda Morrison, Adam G. Gow, Ian Handel, Richard J. Mellanby

**Affiliations:** 1 Royal (Dick) School of Veterinary Studies and The Roslin Institute, The University of Edinburgh, Hospital for Small Animals, Easter Bush Veterinary Centre, Roslin, Midlothian, United Kingdom; 2 Davies Veterinary Specialists Limited, Manor Farm Business Park, Higham Gobion, Herts, United Kingdom; 3 PTDS, Unit 2a, Manor Farm Business Park, Higham Gobion, Herts, United Kingdom; University of Navarra School of Medicine and Center for Applied Medical Research (CIMA), SPAIN

## Abstract

Primary hepatopathies are a common cause of morbidity and mortality in dogs. The underlying aetiology of most cases of canine hepatitis is unknown. Consequently, treatments are typically palliative and it is difficult to provide accurate prognostic information to owners. In human hepatology there is accumulating data which indicates that the presence of systemic inflammatory response syndrome (SIRS) is a common and debilitating event in patients with liver diseases. For example, the presence of SIRS has been linked to the development of complications such as hepatic encephalopathy (HE) and is associated with a poor clinical outcome in humans with liver diseases. In contrast, the relationship between SIRS and clinical outcome in dogs with a primary hepatitis is unknown. Seventy dogs with histologically confirmed primary hepatitis were enrolled into the study. Additional clinical and clinicopathological information including respiratory rate, heart rate, temperature, white blood cell count, sodium, potassium, sex, presence of ascites, HE score, alanine aminotransferase (ALT), alkaline phosphatase (ALP), bilirubin and red blood cell concentration were available in all cases. The median survival of dogs with a SIRS score of 0 or 1 (SIRS low) was 231 days compared to a median survival of 7 days for dogs with a SIRS score of 2, 3 or 4 (SIRS high) (p<0.001). A Cox proportional hazard model, which included all other co-variables, revealed that a SIRS high score was an independent predictor of a poor clinical outcome. The effect of modulating inflammation on treatment outcomes in dogs with a primary hepatitis is deserving of further study.

## Introduction

Primary hepatopathies are a common cause of morbidity and mortality in dogs, with a recent study reporting that over 10 per cent of dogs had evidence of chronic hepatitis at post mortem examination [1–3]. Primary hepatitis includes all inflammatory disorders of the hepatic parenchyma [4]. In contrast to human medicine, where the type of hepatitis is defined by the inciting cause, few causes of chronic hepatitis have been identified in the dog, and the majority of cases are idiopathic [4]. Although primary hepatitis can be readily diagnosed in dogs through histological examination of a liver biopsy, it remains a challenging and difficult disease to treat [1, 5]. Furthermore, it is difficult to offer clients an accurate prognosis at the time of diagnosis [2].

There is growing evidence that systemic inflammatory response syndrome (SIRS) is a common and serious disorder among human patients with liver diseases [6–8]. It has been suggested that the presence of SIRS can lead to a further deterioration in liver function resulting in significant morbidity and mortality [9, 10]. The presence of SIRS can compromise the function of various organ systems resulting in Multiple Organ Dysfunction Syndrome (MODS) [11]. Hospitalised human patients with cirrhosis and SIRS have more severe hepatic encephalopathy (HE), are more likely to develop hepatorenal syndrome and have non-reversible renal dysfunction [12]. In acute liver failure, the presence of SIRS, whether or not precipitated by infection, has been implicated in the progression of HE, reducing the chances of transplantation and conferring a poorer prognosis [13]. Furthermore, higher SIRS scores are related to the development of acute liver failure in patients with pre-existing hepatitis [14].

There is a clear need to clarify the relationship between inflammation and outcome in dogs with a primary hepatitis. A more detailed understanding of the relationship between inflammation and clinical outcomes may lead to novel therapeutic approaches. The aim of this study was to determine the prevalence of systemic inflammation in a cohort of dogs with primary hepatitis, and to then examine the relationship between systemic inflammation and patient survival.

## Materials and Methods

Consecutive cases of dogs diagnosed with primary hepatopathies at the Royal (Dick) School of Veterinary Studies or Davies Veterinary Specialists, were considered for inclusion in the study. Cases were only included if the dog had histopathological confirmation of primary parenchymal hepatic disease. Information on the signalment and previous medical treatment was extracted from the clinical records. The histopathological diagnosis was classified according to WSAVA (World Small Animal Veterinary Association) criteria by a board certified pathologist [4]. All blood samples were taken by a veterinary surgeon as part of routine clinical management of each patient. The study was approved by The University of Edinburgh Ethics Research Committee and the owners of the dogs gave permission for their animals’ data to be used in this study.

The presence of HE was evaluated using previously described criteria [15]. The dog was considered to have HE if it had clinical signs of lethargy, inappropriate behavior, disorientation, circling, head pressing or seizures [16]. If the dog displayed none of these signs, it was considered not to have HE. The presence of ascites was documented if confirmed by ultrasound evaluation. Details of heart rate, respiratory rate, temperature, haematology profile and biochemistry profile were recorded. A SIRS score was calculated for each dog using methodology previously described [17]. The SIRS score could range from 0–4 as each dog was given 1 point when they met each of the following criteria: respiratory rate greater than 20 min heart rate greater than 120 min; total white blood cell (WBC) count less than 6 or greater than 16 x10^9^ L and rectal temperature less than 38.1°C or greater than 39.2°C Therefore, a SIRS score could range from 0 to 4.

Survival of the cohort was initially examined using Kaplan Meier analysis. As some of the dogs were alive at the end of follow up, Cox proportional hazard analysis was used to estimate the association between survival and selected co-variates. Initially, a uni-variable analysis was performed which explored the relationship between survival and SIRS score. To determine if this relationship persisted in the presence of other possible other confounding variables, a multivariable Cox proportional hazard model was constructed which included SIRS score of 0 or 1 (SIRS low) or SIRS score of 2, 3 or 4 (SIRS high), sodium, potassium, sex, ALT, ALP, bilirubin, red blood cell count, presence of ascites, age and HE score. A final model was built based on step wise removal of variables from the initial comprehensive model using AIC as a measure of parameter penalised model fit. To confirm the model structure, the impact on AIC of both dropping each of the final variables and adding back in the dropped variables was investigated. Results are presented as a hazard ratio with 95% CI. Statistical analysis was performed in R statistical software package (R Development Core Team (2012)).

## Results

**Fig 1:**
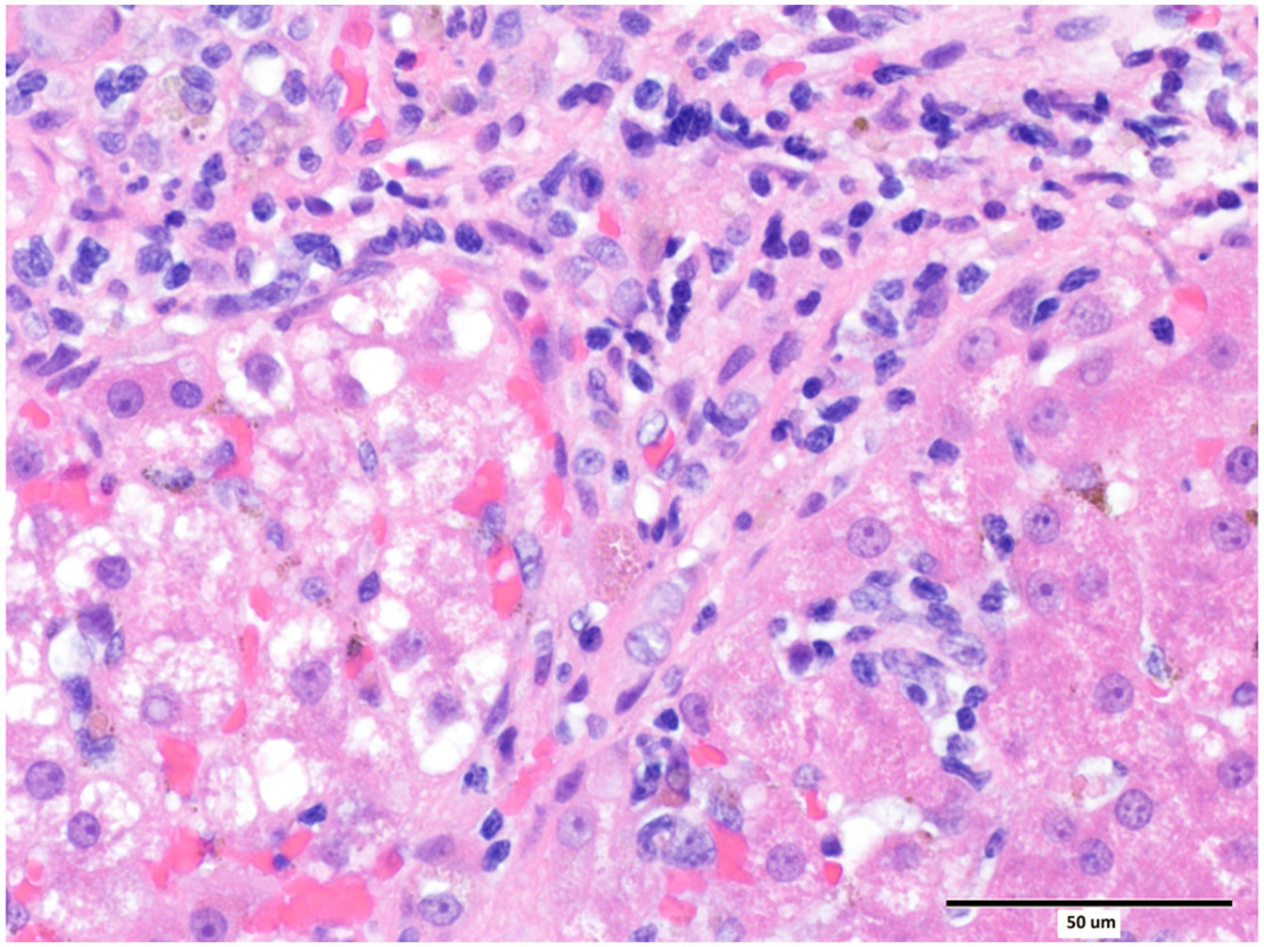
Liver from a case of chronic hepatitis with islands of hepatocytes separated by bands of fibroblasts and collagen, and moderate numbers of macrophages, lymphocytes and plasma cells. Haematoxylin and eosin, scale bar = 50 μm.

**Fig 2:**
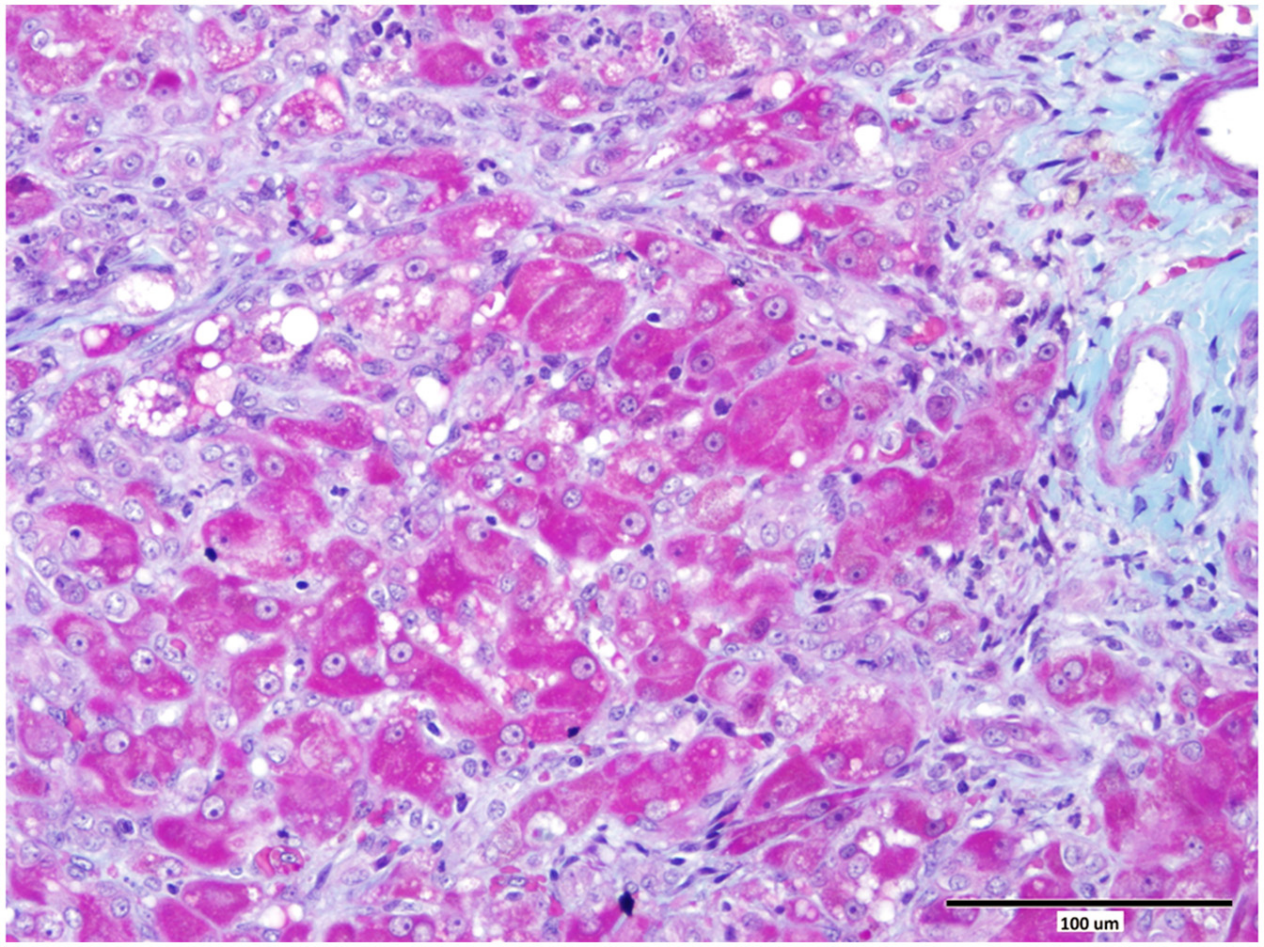
Liver from a case of chronic hepatitis and early cirrhosis showing disruption of the lobular architecture and dissecting fibrosis (green). Masson’s trichrome, scale bar = 100 μm.

**Fig 3:**
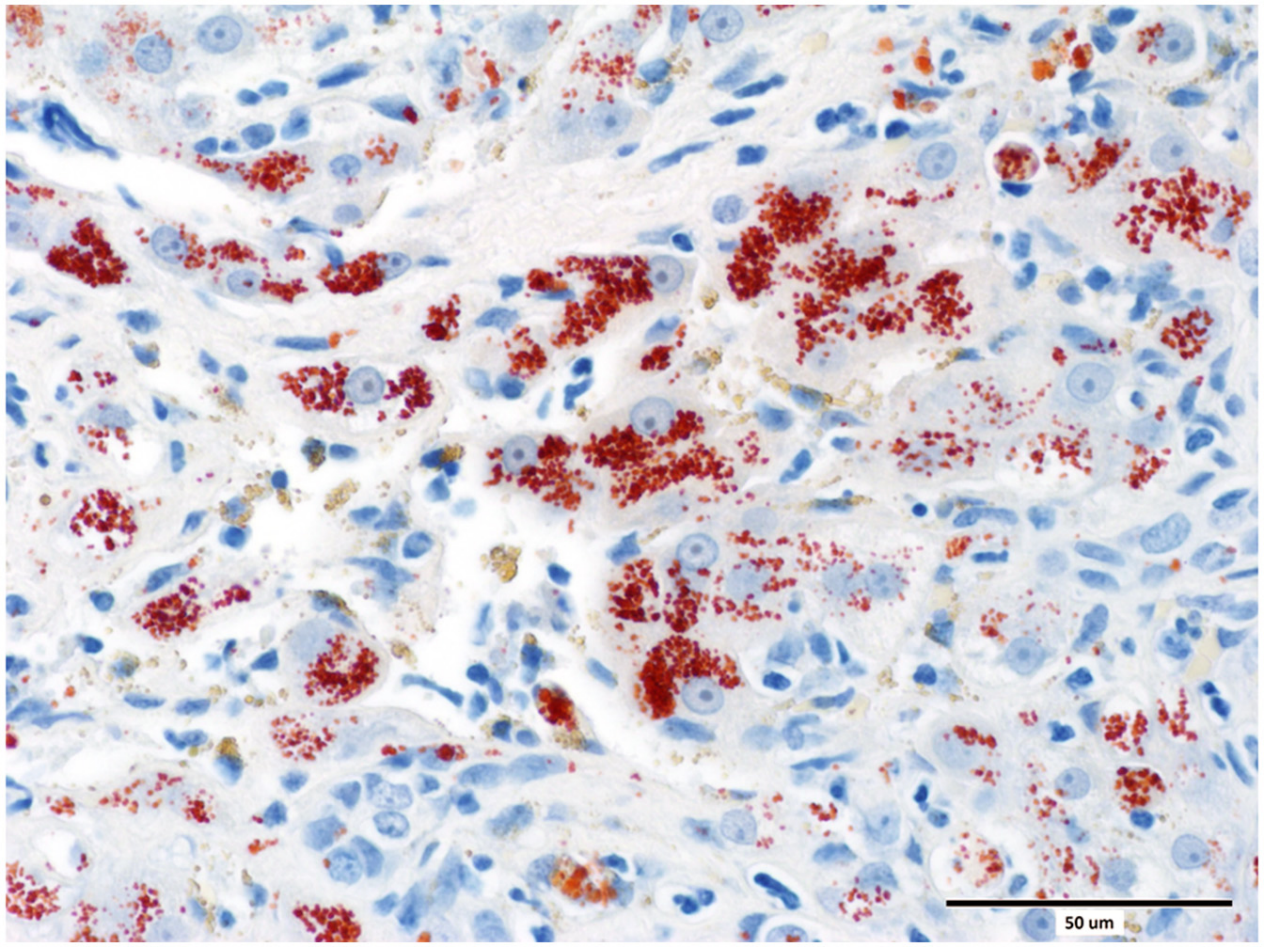
Liver from a case of chronic hepatitis with intracytoplasmic copper accumulation in hepatocytes. Rhodanine red, scale bar = 50 μm.

Eighty cases of histologically confirmed primary hepatopathies were identified. Ten dogs were excluded from the study due to incomplete clinical records. The 70 dogs included in this study comprised of 29 different pure breeds and eight cross-breeds. The sex distribution comprised of 12 entire males, 24 neutered males, 24 neutered females and 10 entire female dogs. The median age was 75 months (range 6 to 180 months). The diagnosis of hepatitis was histologically confirmed from samples collected at post-mortem examination in 9 cases, and from a percutaneous spring-loaded biopsy instrument in 12 cases. Forty nine samples were collected by surgical biopsy techniques. Fifty cases were diagnosed with chronic hepatitis (Fig 1), 10 cases with acute hepatitis, eight cases with cirrhosis (Fig 2) and two cases with copper associated hepatitis (Fig 3). Fifty-seven dogs were dead at follow up and 13 were alive. Seventeen dogs had a SIRS score of 0, 22 had a score of 1, 25 had a score of 2, five had a score of 3 and one dog had a score of 4.

**Fig 4:**
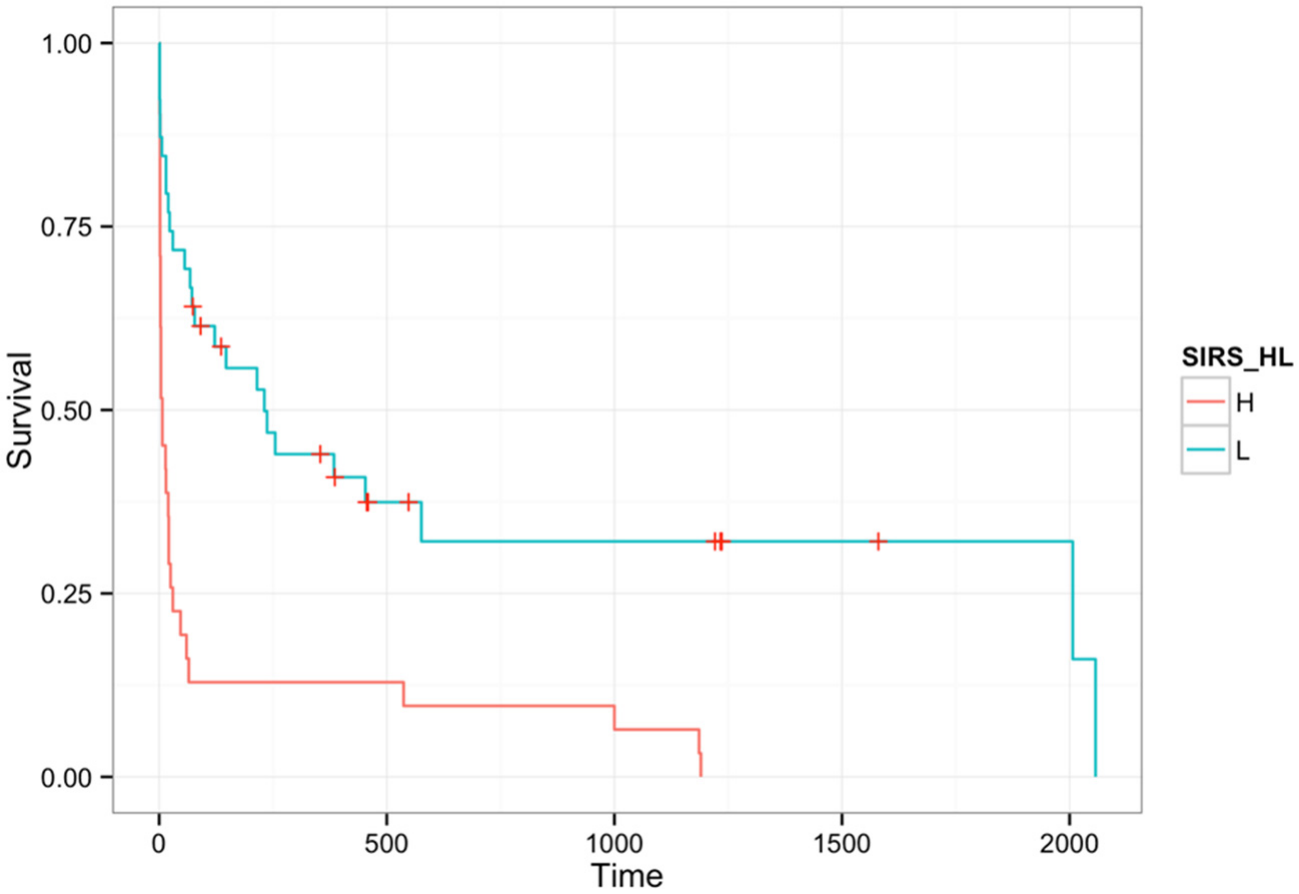
Kaplan-Meier plot of survival time in days by SIRS high (red solid line) and SIRS low (blue dotted line). Tick marks show censored events.

The median survival time of the 70 dogs was 38.5 days (95% CI 20–215). To explore the relationship between a systemic inflammatory response and survival, the median survival of dogs with a SIRS low and dogs with a SIRS high was estimated. The median survival of SIRS low dogs was 231 days (95% CI 78-not available/ days) and median survival of SIRS high was 7 days (95% CI 3-25/ days) (p<0.001) (Fig 4). To assess whether this relationship persisted in the presence of other co-variates, a Cox proportional hazard model was built. The initial model included SIRS high or low, presence of ascites, HE score, sex, sodium, potassium, ALT, ALP, bilirubin, age, sex and red blood cell count (S1 File). The final model after variable selection included SIRS, bilirubin, red blood cell concentrations and age (Table 1). The impact of variables dropped from the model was confirmed by calculating the impact on model fit (Δ AIC) when they were individually added back into the model (Table 2).

**Table 1:** Hazard Ratios and 95% confidence interval for terms in final Cox proportional hazard model. Δ AIC is the increase in AIC if the term is dropped from the model. A positive Δ AIC equates to a poorer model fit.

	Hazard Ratio (95% CI)	Δ AIC
SIRS	3.575 (1.857–6.884)	13.3
Bilirubin	1.003 (1.001–1.005)	5.98
Red blood cell	0.768 (0.596–0.990)	2.14
Age	0.909 (0.827–1.000)	1.94

**Table 2:** Δ AIC for terms not included in final model when added back in final model. A positive Δ AIC equates to a poorer model fit.

	Δ AIC
Sodium	0.03
Potassium	1.77
Sex	2.75
Ascites	1.94
HE score	4.77
ALP	1.98
ALT	0.68

## Discussion

In this large two centre study, we have examined for the first time, the relationship between SIRS, a simple marker of inflammation, and clinical outcome in dogs with primary hepatitis The central finding of this study was that the dogs with a primary hepatitis and a SIRS score of 2, 3 or 4 had a significantly worse clinical outcome than dogs with a SIRS score of 0 or 1. This provides evidence that the presence of systemic inflammation is associated with a poorer clinical outcome in dogs, which is similar to human patients with liver disease. The presence of SIRS was a major predictor of multiple organ failure and strongly correlated with short term mortality in patients with alcoholic hepatitis [7]. In addition, SIRS has recently been associated with acute on chronic liver failure (ACLF) [18].

Inflammation may be associated with a poor outcome in dogs and humans with liver disease for several reasons. Intestinal bacterial overgrowth and altered gut permeability in patients with liver disease is hypothesised to lead to increased translocation of bacteria and endotoxin into the portal circulation [9]. Factors leading to SIRS development in liver disease include the impaired phagocytic function of the reticuloendothelial system which allows endotoxin to reach the systemic circulation in high concentrations [13]. Lipopolysaccharide (LPS) concentrations in the peripheral and portal circulation have been shown to be increased in people with liver cirrhosis. Importantly, LPS concentrations have been shown to predict mortality in patients with alcoholic hepatitis[7]. Endotoxin can activate monocytes and release pro-inflammatory cytokines which have been implicated in the development of hepatic complications such as HE, ascites and variceal bleeding [19]. The disturbances in systemic and hepatic haemodynamics in alcohol-related liver failure (ACLF) have also been associated with dysregulated inflammation, multi-organ failure and marked activation of the sympathetic nervous system [7]. These abnormalities predict high mortality rates in these patients [8].

In our study, the proportion of dogs which had an infection was not accurately defined. In a recent study of human patients with alcoholic hepatitis and SIRS, over 60% of patients had no evidence of infection, based on clinical or microbiological criteria [8]. Understanding the pathophysiology of sterile inflammation in patients with liver disease, with the ambition of developing targeted therapies, is one of the major challenges in contemporary hepatology.

Predicting the course and prognosis of disease is an important part of veterinary and human clinical practice and the development of new prognostic markers with documented significance is an important focus of human and veterinary research. Various factors have been demonstrated as negative prognostic indictors in dogs with primary hepatitis [20]. These include jaundice, abdominal fluid wave, microhepatica, ascites, enlarged portal lymph nodes, hypoalbuminemia and left shift of neutrophils in the peripheral blood [1]. Consistent with our study, total serum bilirubin was a negative prognostic indicator in idiopathic canine chronic hepatitis [2]. High bilirubin concentrations have also been documented to be associated with a negative outcome in human patients [21, 22]. Furthermore, our prognostic link with low red blood cell count is consistent with the prediction of poor post-surgical survival associated with reduced red blood cell counts in human patients with primary liver cancer [23] and end stage liver disease [24].

Another finding of this study was that abnormal serum sodium concentrations were not commonly observed in dogs with primary hepatitis. This observation contrasts with the findings from studies of humans with liver disease where hyponatremia is frequently observed [25–27]. For example, in a prospective study of 997 patients with cirrhosis the prevalence of low serum sodium concentration, defined as a serum sodium ≤135 mmol/l, ≤130 mmol/l, ≤125 mmol/l or ≤120 mmol/l, was 49.4%, 21.6%, 5.7%, and 1.2%, respectively [28]. Borroni et al. reported hyponatraemia in 29.8% of patients admitted for management of cirrhosis, as defined by a serum sodium concentration of ≤130 mmol/l [29]. Furthermore, the prevalence of hyponatremia was 26% in a population of paediatric patients with end stage liver disease awaiting liver transplantation (as defined by a serum sodium concentration of ≤130 mmol/l) [30]. It remains unclear why humans frequently develop hyponatraemia with liver disease while dogs do not. We hypothesise that the differences may reflect the different causes of liver disease in dogs compared to humans.

Several studies in human patients with liver disease have examined the role of circulating biomarkers as adjunctive markers of systemic inflammation, such as LPS, procalcitonin and C reactive protein [31–33]. Cytokines, including interleukin-6 (IL-6), interleukin-8 (IL-8) and tumour necrosis factor (TNF), are key mediators of the inflammatory response [11]. Several studies have measured circulating cytokine concentrations in patients with liver disease. One marker that has been investigated in some detail is IL-6 [34–36]. Increased serum concentrations of IL-6 have been found in people with different inflammatory diseases and has been correlated with the severity of disease, prognosis and outcome [37]. Higher circulation levels of IL-6 were significantly associated with a higher risk of developing hepatocellular carcinoma [38]. IL-6 has also been shown to be a useful prognostic marker for canine critical care patients [39].

Our study demonstrates the need to understand more about the development of systemic inflammation in dogs with liver disease and to establish whether reducing systemic inflammation improves outcomes.

## Conclusion

In conclusion, a high SIRS score was associated with poorer long term survival in our study of dogs with primary hepatitis Our study demonstrates the need to understand more about the causes and consequences of inflammation in dogs with liver disorders. Our study demonstrates the need to understand more about the development of systemic inflammation in dogs with liver disease and to establish whether reducing systemic inflammation improves patient outcomes. The impact of ameliorating inflammation in patients with hepatitis is ill defined. Although prednisolone is widely used as a first line treatment for hepatitis in both dogs and humans, its therapeutic benefits remain ill-defined in both species [40–43].

## Supporting Information

S1 File Clinical and biochemical data from the 70 dogs with histologically confirmed hepatitis reported in this study. (XLSX)
